# Acute and delayed mild coagulopathy are related to outcome in patients with isolated traumatic brain injury

**DOI:** 10.1186/cc9399

**Published:** 2011-01-05

**Authors:** Sjoerd Greuters, Annelies van den Berg, Gaby Franschman, Victor A Viersen, Albertus Beishuizen, Saskia M Peerdeman, Christa Boer

**Affiliations:** 1 Department of Anesthesiology, Institute for Cardiovascular Research, VU University Medical Center, De Boelelaan 1117, 1081 HV Amsterdam, The Netherlands; 2 Department of Intensive Care Medicine, Institute for Cardiovascular Research, VU University Medical Center, De Boelelaan 1117, 1081 HV Amsterdam, The Netherlands; 3 Department of Neurosurgery, VU University Medical Center, De Boelelaan 1117, 1081 HV Amsterdam, The Netherlands

## Abstract

**Introduction:**

The relationship between isolated traumatic brain injury (TBI) associated coagulopathy and patient prognosis frequently lacks information regarding the time course of coagulation disorders throughout the post-traumatic period. This study was conducted to assess the prevalence and time course of post-traumatic coagulopathy in patients with isolated TBI and the relationship of these hemostatic disorders with outcome.

**Methods:**

The local Human Subjects Committee approved the study. We retrospectively studied the medical records of computed tomography (CT)-confirmed isolated TBI patients with an extracranial abbreviated injury scale (AIS) <3 who were primarily referred to a Level 1 trauma centre in Amsterdam (*n *= 107). Hemostatic parameters including activated partial thromboplastin time (aPTT), prothrombin time (PT), platelet count, hemoglobin, hematocrit, glucose, pH and lactate levels were recorded throughout a 72-hour period as part of a routine standardized follow-up of TBI. Coagulopathy was defined as a aPPT >40 seconds and/or a PTT in International Normalized Ratio (INR) >1.2 and/or a platelet count <120*10^9^/l.

**Results:**

Patients were mostly male, aged 48 ± 20 years with a median injury severity score of 25 (range 20 to 25). Early coagulopathy as diagnosed in the emergency department (ED) occurred in 24% of all patients. The occurrence of TBI-related coagulopathy increased to 54% in the first 24 hours post-trauma. In addition to an increased age and disturbed pupillary reflex, both coagulopathy upon ED arrival and during the first 24 hours post-trauma provided an independent prognostic factor for unfavorable outcome (odds ratio (OR) 3.75 (95% CI 1.07 to 12.51; *P *= 0.04) and OR 11.61 (2.79 to 48.34); *P *= 0.003).

**Conclusions:**

Our study confirms a high prevalence of early and delayed coagulopathy in patients with isolated TBI, which is strongly associated with an unfavorable outcome. These data support close monitoring of hemostasis after TBI and indicate that correction of coagulation disturbances might need to be considered.

## Introduction

Acute coagulopathy in the absence of extracranial injuries is a severe complication of traumatic brain injury (TBI) and may contribute to secondary injury and mortality [1,2]. Among others, brain injury is associated with activation of the coagulation cascade through fulminant cerebral tissue factor release, contributing to disseminated intravascular coagulation and cerebral microthrombi. This process is independent of bleeding [3-6]. The subsequent disparity between clot formation and fibrinolysis in combination with coagulopathy may increase the risk for delayed or secondary bleeding [7,8]. A recent meta-analysis showed an overall prevalence of TBI-associated coagulopathy of 33% and a strong relationship of hemostatic disorders with unfavorable outcome in these patients [9]. However, the prevalence of coagulopathy in isolated TBI differed considerably among studies due to the variety in study design and definitions of traumatic coagulopathy [9].

Early recognition of coagulopathy is of value in predicting the occurrence of delayed brain injury and may contribute to prevention of bleeding disorders [10]. Most studies, however, report a mixture of early and delayed coagulopathy in isolated TBI, and knowledge about the prognostic value of acute, early diagnosed coagulopathy is therefore limited. A recent evaluation of a large German trauma registry revealed that 23% of patients with isolated TBI are presented with acute coagulopathy upon emergency department (ED) arrival, which was associated with increased morbidity and mortality [11]. Although the prevalence of coagulopathy increases in the period after ED admission, there are only limited data available about the evolvement of hemostatic parameters and the relative number of patients that develop delayed coagulopathy in the first days post-trauma [12-14].

The aim of the present study was to investigate the incidence of early and delayed coagulopathy in patients with isolated TBI and an extracranial Abbreviated Injury Score less than three. Furthermore, we evaluated the progression of coagulopathy in the first 72 hours post-trauma and the predictive value of TBI-related early coagulopathy with outcome in addition to prognostic factors like age, the Glasgow Coma Scale and pupillary reflex.

## Materials and methods

### Patient population

This retrospective evaluation comprised patients with traumatic brain injury (TBI) who were primarily admitted to the Emergency Department of the VU University Medical Center Amsterdam in the Netherlands during the period 2003 to 2007. The Local Human Subjects Committee of the VU University Medical Center approved the study and waived the requirement to obtain informed consent. Data were retrieved from the Amsterdam Lifeliner: Analysis of Results and Methods (ALARM) database which was based on the electronic hospital admission register and patient medical records [15]. Isolated TBI was defined as CT-scan confirmed brain tissue injury without other major injuries as indicated by an Abbreviated Injury Score (AIS) <3. The reported Glasgow Coma Scale ranged from 3 to 8 (severe TBI), 9 to 13 (moderate TBI) and 14 to 15 (mild TBI). Exclusion criteria were an extracranial AIS score of 3 or more, age <16 years, use of coumarins, liver failure or missing coagulation parameters on hospital admission.

### Data collection and definitions

Patients' records were evaluated for the following variables: trauma mechanism, use of anticoagulants, age, sex, reported Glasgow Coma Scale (GCS) upon ED arrival, injury severity score (ISS), abbreviated injury score (AIS), pupil reflexes, need for acute surgery, volume of pre-hospital administered intravenous fluid, the administration of tranexamic acid, fresh frozen plasma (FFP), platelets, packed cells and TBI-related mortality in the post-trauma period. Furthermore, the activated partial thromboplastin time (aPTT), the international normalized ratio (INR) in the prothrombin time (PT), platelet count, hemoglobin, hematocrit, glucose, pH and lactate levels were recorded until 72 hours after the trauma. The first blood samples were immediately drawn after arrival at the ED, whereas subsequent samples were drawn at regular time points after trauma. Funding of our Level 1 trauma center requires ISS calculation for every admitted trauma patient by our trauma database registration officer, and these ISS values were re-checked by an independent researcher. Moreover, two physicians independently calculated the AIS value based on the final diagnosis after hospital admission. A disturbed pupil reflex was defined as a uni- or bilateral failure of pupil reflexes. Coagulopathy was defined as an aPTT >40 seconds and/or a PTT in INR >1.2 and/or a platelet count <120*10^9 ^per liter.

### Statistical analysis

Data were analyzed using SPSS 16.0 (SPSS Inc, Chicago, IL, USA). Parametric data like age and laboratory values were represented as mean with SD or SEM whereas non-parametric data like the GCS were shown as median with interquartile ranges (IQR), respectively. Patient en clinical characteristics were analyzed with a Student's *t*-test, a Mann-Whitney U-test or a Chi-square test for continuous normally distributed, nonparametric continuous, and dichotomous data, respectively. Multinomial regression analysis was performed to evaluate the prognostic value of early coagulopathy upon admission to the ED, age, GCS and pupillary reflex for patient survival. A *P*-value of <0.05 was considered significant.

## Results

The total database consisted of 247 patients, of which 107 subjects were eligible for inclusion for data analysis. Reasons for exclusion were an extracranial AIS score >3 (*n *= 76), age <16 years (*n *= 41), use of coumarins (*n *= 9), liver failure (*n *= 3), missing coagulation parameters at admission (*n *= 8) and miscellaneous reasons (*n *= 3). Patients with isolated TBI were typically male (74%) and aged 48 ± 20 years with a median ISS of 25 (range 20 to 25). In the total patient group, 65% suffered from severe TBI, whereas moderate and mild TBI were reported in 18% and 17% in the population, respectively.

### Coagulopathy in isolated TBI upon ED arrival

The general characteristics of patients with (*n *= 26) or without (*n *= 81) coagulopathy upon ED admission are represented in Table 1. The incidence of coagulopathy upon ED arrival associated with brain tissue injury estimated 24%. Baseline parameters including age, gender, ISS, GCS, type of trauma, involvement of a physician-based emergency medical service (EMS), transportation time and need for acute surgery were similar for both groups. Patients with coagulopathy more frequently showed a disturbed pupil reflex (50% vs. 28%; *P *= 0.04) and received more pre-hospital fluid resuscitation (0.98 ± 1.00 liter vs. 0.53 ± 0.55 liter; *P *= 0.01) as compared to patients without early hemostatic disorders. Moreover, patients without coagulopathy more frequently used alcohol before the traumatic incident. There were no differences in the use of fraxiparin, clopidogrel or aspirin between both groups.

**Table 1:** Admission characteristics of patients with isolated TBI with and without coagulopathy upon emergency department arrival

	Coagulopathy	No coagulopathy	*P*-value
**N**	26	81	
**Age (years)**	49 ± 22	47 ± 19	n.s.
**Males**	65%	76%	n.s.
**ISS (median)**	25 (10 to 29)	25 (9 to 43)	n.s.
**GCS (median)**	3 (3 to 15)	6 (3 to 15)	n.s.
**Disturbed pupil reflex**	50%	28%	0.04
**Blunt trauma**	92%	94%	n.s.
**Involvement physician-based EMS**	40%	34%	n.s.
**Intubated at the trauma scene**	38%	30%	n.s.
**Pre-hospital fluid resuscitation (liter)**	0.98 ± 1.00	0.53 ± 0.55	0.01
**Time from injury to ED (minutes)**	47 ± 19	57 ± 43	n.s.
**Need for acute surgery**	73%	80%	n.s.
**Alcohol**	9%	29%	0.04
**Fraxiparin**	4.2%	1.4%	n.s.
**Clopidogrel**	0%	1.4%	n.s.
**Asprin**	20.8%	8.3%	n.s.

ED, emergency department; EMS, emergency medical service; GCS, Glasgow Coma Score; ISS, injury severity score; n.s., not significant. Values represent percentages, mean ± SD or median with interquartile range.

Table 2 shows the laboratory values upon ED admission of patients with and without coagulopathy. Patients with coagulopathy had lower hemoglobin levels, platelet counts, and an increased PT and aPTT in comparison with patients without coagulopathy, while there were no differences in pH and lactate among groups.

**Table 2:** Hemostatic parameters of patients with isolated TBI with and without coagulopathy

	Coagulopathy	No coagulopathy	*P*-value
**N**	26	81	
**Hemoglobin (mmol/l)**	7.4 ± 1.6	8.4 ± 0.8	<0.001*
**PT**	1.4 ± 0.6	1.1 ± 0.1	<0.001*
**aPTT (s)**	46 ± 29	31 ± 4	<0.001*
Platelets (*10^**9 **^**per liter)**	180 ± 53	241 ± 61	<0.001*
**pH**	7.34 ±0.10	7.37 ± 0.10	n.s.
**Lactate (mmol/l)**	2.8 ± 1.8	2.3 ± 1.2	n.s.
**Coagulopathy at 24 h post-trauma [n]**	26/26 (100%)	32/81 (40%)	-

aPTT, activated partial thromboplastin time; n.s., not significant; PT, prothrombin time. Values represent percentages, mean ± SD or median with interquartile range.

### The time course of hemostasis in isolated TBI

**Figure 1:**
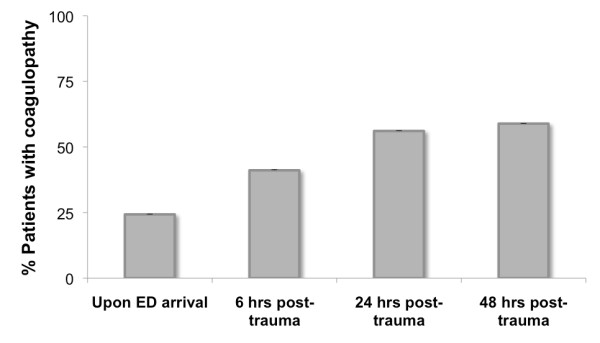
**The relative number of patients with a diagnosis of coagulopathy**. The relative number of patients with a diagnosis of coagulopathy upon emergency department arrival and at 6, 24 and 48 hours post-trauma.

Figure 1 shows the percentage of patients with a coagulopathy upon ED arrival and at 6, 24 and 48 hours post-trauma. In the group of patients without hemostatic alterations upon ED arrival, 40% developed a delayed coagulopathy in the first 24 hours post-trauma (Table 2). In 14 subjects without signs of post-traumatic coagulopathy at ED admission, hemostatic disorders became evident during surgery and resulted in the perioperative administration of blood products.

### The relationship between coagulopathy and outcome in isolated TBI

Multinomial regression analysis was performed in all patients with complete datasets for age, GCS category, pupillary reflex, and coagulopathy upon ED arrival (*n *= 102). Table 3 presents the odds ratios for patients who died (*n *= 34) or survived (*n *= 68) after isolated TBI. In agreement with international literature, age and pupillary reflex affected the mortality probability by 1.07 and 4.78, respectively (both *P *< 0.05). The GCS category had no predictive value in this specific analysis. Coagulopathy upon ED admission was predictive for patient outcome (OR 3.75; *P *= 0.04). Moreover, multinomial regression including age, pupillary reflex and GCS category revealed even a stronger prognostic value for the occurrence of coagulopathy in the first 24 hours post-trauma (OR 11.61 (95% CI 2.79 to 48.34); *P *= 0.003).

**Table 3:** Presentation of odds ratios for patients who died or survived after isolated TBI

	Died	Survived	OR (95% CI)	*P*-value
**Valid cases (n)**	34	68		
**Early coagulopathy**				
*No coagulopathy*	20 (59%)	57 (84%)		
*Coagulopathy*	14 (41%)	11 (16%)	3.75 (1.07 to 13.51)	0.04*
**Age (years)**	59 ± 17	42 ± 19	1.07 (1.03 to 1.11)	0.001*
**GCS category [n]**				0.04*
*<9*	31 (91%)	35 (51%)	7.11 (0.60 to 84.53)	0.12
*9 to 13*	2 (6%)	16 (24%)	2.28 (0.14 to 37.72)	0.56
*>13*	1 (3%)	17 (25%)		
**Pupillary reflex (n)**				
*Normal pupillary reflex*	11 (35%)	55 (81%)		
*Disturbed pupillary reflex*	23 (65%)	13 (19%)	4.78 (1.35 to 17.00)	0.02*
Nagelkerke R^2 ^= 0.61				

Values are represented as number of patients or mean ± SD. In case of patient numbers, percentages were calculated for both groups (died versus survived). *P < 0.05.

## Discussion

The present study showed that early coagulopathy was prevalent in a quarter of patients with isolated traumatic brain injury, and this number doubled in the first 24 hours post-trauma. These data are consistent with findings in more than 3,000 patients with isolated TBI in a large German Trauma Registry [11]. In addition to patient age and pupillary reflexes, coagulopathy upon ED arrival and at 24 hours post-trauma was predictive for unfavorable outcome in patients with closed head injury. The strong prognostic value of coagulopathy in the first 24 hours post-trauma warrants early and recurrent coagulation monitoring in isolated head injury.

In contrast to other studies [16], we showed no significant relation between GCS and patient outcome, although the odds ratio for GCS was high. Our trauma care system recommends pre-hospital endotracheal intubation in all patients with severe TBI, which results in a relatively high number of endotracheally intubated patients admitted to the ED. The low GCS value associated with endothracheal intubation upon ED arrival may blur the predictive value of the level of brain injury for patient prognosis.

The pathophysiological mechanism underlying coagulopathy in isolated TBI is multifactorial and still subject of debate. It is thought that isolated TBI induces massive tissue factor release into the general circulation, which may lead to traumatic intravascular coagulation abnormalities [2-7,17,18]. Moreover, an imbalance between clot formation and hyperfibrinolysis as a result of systemic hypoperfusion and anticoagulation may subsequently contribute to consumption coagulopathy and bleeding disorders [5,7]. Hypoperfusion promotes endothelial thrombomodulin expression that binds thrombin, thereby inhibiting fibrin generation from fibrinogen. Moreover, the thrombomodulin-thrombin complex additionally activates protein C, which inhibits plasminogen activator inhibitor 1 (PAI-1) and the coagulation factors Va and VIIIa. Simultaneously, endothelial t-PA release contributes to the initiation of fibrinolysis [5,7]. Additionally, TBI patients frequently suffer from hypothermia and acidosis, which both contribute to deterioration of the hemostasis and impaired outcome [18-21].

The prevalence of coagulopathy in isolated TBI varies considerably among studies and proportions between 10% and 90% have been reported [9]. This variation may be ascribed to various reasons, including inconsistency in the definition of coagulopathy, diversity in the level of injury severity among studies and the mixture of early and delayed hemostatic disorders to calculate the prevalence of isolated TBI-associated coagulopathy. First, the definition of traumatic coagulopathy in isolated TBI differs between studies, and evidence-based guidelines for the definition of TBI-related hemostatic disorders are lacking. Furthermore, TBI-associated coagulopathy is frequently not related with visual blood loss, whereas most strategies focus on the volume of blood loss as an indicator of traumatic coagulopathy. In the last decade, disseminated intravascular coagulation (DIC) has been proposed as an indicator of early TBI-related coagulopathy by the International Society on Thrombosis and Haemostasis, and the diagnostic criteria for DIC have recently been simplified [22,23]. However, the DIC score has only been scarcely used to diagnose isolated TBI-related coagulopathy, and most studies rely on classical laboratory parameters like the activated partial thromboplastin time (aPTT), the prothrombin time (PT), the international normalized ratio (INR) in the PT, fibrinogen levels and platelet count. Olson *et al*. earlier reported that mild alterations in the aPTT (>34 seconds) and platelet count (<150*10^9 ^per liter) may already be indicative for early coagulopathy in isolated head injury [1]. Others reported aPTT and PT values ranging from 34 to 60 and 13 to 18 seconds, respectively, and an INR of >1.1 to 1.5 and platelet counts of 100 to 150*10^9^/μl as definition for coagulopathy associated with isolated TBI [1,12,13,23-25]. The definition of coagulopathy as used in the present study was in agreement with the abovementioned ranges. However, since coagulopathy-related outcome prediction in isolated TBI depends on the definition of coagulopathy, the development of evidence-based guidelines is warranted.

The number of isolated TBI patients with coagulopathy doubled in the first 24 hours after trauma in our cohort. Carrick *et al*. earlier showed that patients with moderate and severe TBI are at risk for the development of coagulopathy, not only at admission, but also on subsequent laboratory investigation [12]. Interestingly, our data showed that all patients with early hemostatic disorders also met our criteria for coagulopathy after 24 hours post-trauma. Moreover, 40% of patients without early coagulopathy developed hemostatic abnormalities in the first day after admission to our ED. In our patient population, hemostatic evaluation in isolated TBI patients started upon hospital admission, but in some patients who developed late coagulopathy there were no serial laboratory examinations performed. Our data, however, show that hemostatic abnormalities may continue until the third day post-trauma and should be carefully monitored. Indeed, it has been reported that hemostatic abnormalities may be observed until six days post-trauma [12,26]. These findings, in combination with the strong predictive value of early or late coagulopathy for outcome in isolated TBI, suggest that serial hemostatic evaluations in isolated TBI should be included in standard trauma protocols. Our study was limited by the absence of hemostatic laboratory values like ionized calcium, fibrinogen, D-dimer, protein C levels or thromboelastographic dynamics. Furthermore, this study lacks information regarding the core body temperature.

In a large German trauma registry it has been shown that the volume of plasma expanders administered in the pre-hospital period is associated with the occurrence of early traumatic coagulopathy [27]. Despite the higher volume of fluid administration in patients with isolated TBI-associated coagulopathy as compared to subjects without hemostatic disorders, this was no predictor of patient outcome. Recent findings suggest that pre-hospital fluids exceeding 2,000 ml may independently be associated with coagulopathy in patients with isolated blunt TBI [28]. In our trauma region, prehospital fluid administration frequently does not exceed a volume of 1,000 ml, which might explain the absent relationship between pre-hospital fluid administration and coagulopathy in our patient population. Additionally, our data confirmed that alcohol intoxication is a common finding in patients with isolated TBI and suggested that alcohol intoxication is associated with a lower incidence of coagulopathy as compared to non-intoxicated patients [28].

Early hemostatic monitoring, including point-of-care thromboelastography or thromboelastometry, may contribute to early diagnosis of coagulation disorders in patients with isolated head injury. Although data regarding blood transfusion in early and late coagulopathy in traumatic brain injury are limited, an increasing number of investigations focus on the administration of recombinant factor VII as primary therapy for isolated TBI-related coagulopathy [29-31]. Further studies are needed to investigate optimal treatment strategies in patients with early or delayed coagulopathy following isolated head injury.

## Conclusions

The present study shows that patients with isolated TBI are at risk for the development of acute and delayed coagulopathy, which is strongly associated with poor patient prognosis. Based on our results we emphasize the importance of early diagnosis of coagulation in isolated TBI patients. Routine determination of coagulation parameters is warranted, especially in the first 24 hours post-trauma. Future studies should reveal whether early recognition of acute coagulopathy and prevention of delayed hemostatic disturbances might be associated with improvement of morbidity and mortality in patients with isolated trauma injury.

## Key messages

• A substantial part of the population with isolated traumatic brain injury is at risk for the development of coagulopathy.

• The number of patients with isolated traumatic brain injury and coagulopathy doubles within 72 hours post-trauma and is closely associated with poor patient prognosis.

• Early diagnosis of coagulopathy in isolated traumatic brain injury may contribute to improved patient outcome.

• Coagulation measurements in severe isolated traumatic brain injury should be frequently performed and continued throughout the first 72 hours after trauma.

## Abbreviations

AIS: abbreviated injury scale; ALARM: Amsterdam Lifeliner: Analysis of Results and Methods; APTT: activated partial thromboplastin time; CI: confidence interval; CT: computer tomography; DIC: disseminated intravascular coagulation; ED: emergency department; EMS: emergency medical service; FFP: fresh frozen plasma; GCS: Glasgow coma scale; FFP: fresh frozen plasma; INR: international normalized ratio; IQR: interquartile range; ISS: injury severity score; PAI-1: plasminogen activator inhibitor; OR: odds ratio; PT: prothrombin time; TBI: traumatic brain injury.

## Competing interests

The authors declare that they have no competing interests.

## Authors' contributions

All authors helped to draft the manuscript or critically revised it. All co-authors agree about the content of the paper and have read the manuscript and approved its submission to Critical Care. Furthermore, SG participated in the design, coordination and data acquisition of the study. AvdB participated in data acquisition, database management and statistical analysis. GF and SP participated in the design of the study. VV and BB participated in data acquisition. CB conceived the study, participated in the design of the study and finalized the manuscript.
